# Supplementary material for: Transcriptome and Metabolome Analysis Revealed That Exogenous Spermidine-Modulated Flavone Enhances the Heat Tolerance of Lettuce
Source: Antioxidants (Basel). 2022 Nov 25;11(12):2332. doi: 10.3390/antiox11122332 (PMC9774108; doi:10.3390/antiox11122332)
Supplement: Supplementary file 1 [file antioxidants-11-02332-s001.zip › SupplementaryMaterialFig1.pdf]

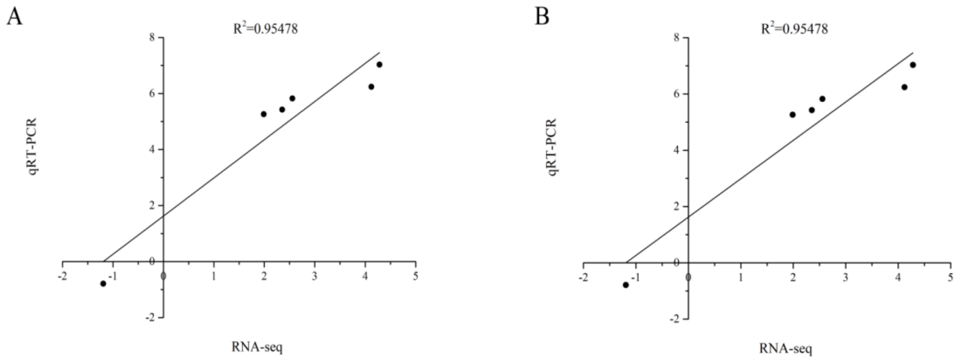

Supplementary Figure S1. Validation of the RNA-Seq results by qRT-PCR. Data are presented as the means of three replicates. (A) HSvsH (B) HvsCK
